# An Atomic-Scale Mechanism of Potassium–Oxygen Redox Chemistry

**DOI:** 10.1021/jacsau.5c00855

**Published:** 2025-09-05

**Authors:** Chao Zhang, Linjie Chen, Jin Zhao, Hrvoje Petek

**Affiliations:** † Hefei National Research Center for Physical Sciences at the Microscale, 12652 University of Science and Technology of China, Hefei, Anhui 230026, China; ‡ Department of Chemical Physics, School of Chemistry, University of Science and Technology of China, Hefei, Anhui 230026, China; § Department of Physics and Astronomy and the IQ Initiative, 6614 University of Pittsburgh, Pittsburgh, Pennsylvania 15260, United States

**Keywords:** scanning tunneling microscopy, density functional theory, surface chemistry, potassium atom, oxygen reduction chemistry, potassium oxide

## Abstract

The interaction between K atoms and oxygen molecules on solid surfaces is of topical interest to oxidation–reduction processes in K–O_2_ batteries. Alkali metals have one *ns* electron in their valence shell, making them highly chemically reactive toward oxidizing reactants. Mechanistic information on the oxygen reduction by K at the atomic level is scarce despite its key role in defining the alkali metal–O_2_ battery performance. Here, we use scanning tunneling microscopy and density functional theory to investigate the reduction of a single oxygen molecule by K atoms codeposited on the Ag(111) surface. Our study provides fundamental chemical information on the binary and collective interactions between the O_2_ and K atoms on metal surfaces.

## Graphical abstract

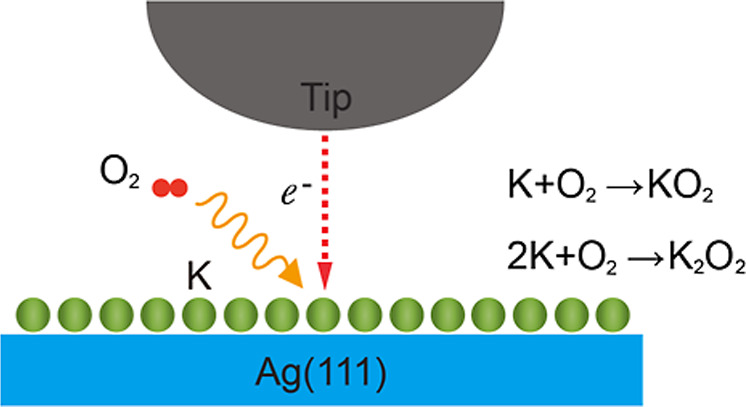

## 1 Introduction

Energy storage is an essential component in the paradigm shift to clean and renewable energy. While the advent of the lithium-ion batteries has deeply penetrated every aspect of our lives, the Li-ion technology based on intercalation chemistry is approaching its performance limits.^1–4^ In an effort to surpass their performance, rechargeable metal–air (more precisely, metal–oxygen) batteries have attracted much interest as their theoretical energy densities are superior to that of the conventional Li-ion batteries.^5–8^ Typical nonaqueous alkali metal–O_2_ (Am–O_2_) batteries that use molecular oxygen redox chemistry at Li^+^/Na^+^/K^+^ cathodes to form alkali superoxides^9–12^ are deemed to be potential alternatives to Li-ion batteries for effective energy storage. Among them, K–O_2_ batteries are of acute interest because of their low overpotentials, high energy efficiencies, elemental earth abundance, and low costs.^8,13,14^ Significantly, the reactivities of potassium, sodium, and lithium atoms toward oxygen molecules are quite different despite their common *ns* valence electron structure.^15–17^ This raises interest regarding the variation of chemical interactions between alkali metal atoms and molecular oxygen, which stimulates our research in exploring the fundamental steps with atomic state resolution in the oxygen reduction chemistry. The design of highly active catalysts and the study of the formation mechanism of the oxide products can lead to improvement of the K–O_2_ battery performance. In particular, how alkali peroxides affect and potentially enhance the performance of Am–O_2_ batteries is an open question.^18,19^

K–O_2_ batteries have higher potential energy density, superior energy efficiency, and a longer lifetime compared with other Am–O_2_ batteries.^18^ Studies have shown that the addition of metals to the carbon cathodes can effectively reduce overpotentials and improve capacity retention.^20,21^ While there are reports on the role of metal catalysts in the oxygen reduction reaction by alkali metals,^22,23^ the precise mechanism underlying the oxygen reduction reaction on the metals remains unclear. Potassium oxides at the molecular scale are known to assume four different chemical compositions: K_2_O, K_2_O_2_, KO_2_, and KO_3_.^15,24^ Formation of such K_*x*_O_*y*_ species has been proposed to occur upon K–O_2_ coadsorption on metal surfaces, including thick K films.^25–29^ Although both experimental and theoretical studies have been performed to elucidate K atom interactions with molecular oxygen on solid surfaces,^30–33^ due to their high complexity, an in-depth atomic-scale understanding of the K–O_2_ interaction on metals is lacking. For instance, fundamental information about the reaction mechanism, the intermediates involved, the oxide product structure, and distribution at the atomic level remains unclear. Studying such processes on simple metal surfaces where they can be imaged and simulated at an atomic scale is an important step toward K–O_2_ battery design, where more complex but difficult-to-elucidate processes play important roles.

Here, we present a study of the redox chemistry of single oxygen molecules with K atoms on the Ag(111) surface by scanning tunneling microscopy (STM) and density functional theory (DFT). While addressing the K–O_2_ battery function by full first-principles simulation of the K–O_2_ battery involves considerable complexity beyond the scope of this work, here we aim instead to establish a benchmark for interpreting the atomic-scale K–O_2_ chemistry observed experimentally by identifying the fundamental interactions that are accessible to interpretation through ab initio theory. We show that at low temperature (∼4.6 K), the partially ionized K adatoms chemisorbed on the Ag(111) surface react with the O_2_ molecules by forming two kinds of ionic complexes (KO_2_ and K_2_O_2_) by partial transfer of some of their remaining 4s electron charge. DFT calculations show that the charge transfer to O_2_ in the K_2_O_2_ complex is larger than those in the KO_2_ complex, and the ionic interactions make K_2_O_2_ complexes more favorable with increasing K atom density. This result is significant for potassium–oxygen batteries, where the KO_2_ complex is thought to be the primary discharge product.^18^ While the role of the K_2_O_2_ peroxide species is not fully understood and may have some practical advantages, it is established that its lithium analog Li_2_O_2_ has sluggish kinetics with severe parasitic reactions related to the singlet oxygen formation.^18,19^ The peroxide–superoxide conversion system is nevertheless of interest because a continuous supply of O_2_ is not necessary in a closed battery system, which could potentially achieve a higher energy density.^34^ Our study provides fundamental information on the interaction between an oxygen molecule and a K atom on the Ag(111) surface for a basic experimental and theoretical understanding of the O_2_–K redox superoxide–peroxide surface chemistry.

## 2 Methods

### 2.1 STM Experiments

The experiments are carried out in a low-temperature ultrahigh vacuum (UHV) scanning tunneling microscopy (Omicron) instrument at a base pressure of ∼1.0 × 10^–10^ Torr. The STM measurements are performed at a sample temperature of ∼4.6 K in the constant current mode. The electrochemically etched tungsten tip is virtually grounded, and a bias voltage is applied to the sample. Clean atomically ordered Ag(111) surfaces are prepared by cycles of argon ion sputtering and annealing. Potassium is deposited at room temperature onto the prepared Ag(111) single crystal surface in a preparation chamber from a commercial dispenser (Alvatec) at a rate of ∼0.01 monolayer (ML) per min (we define an ML as one K atom per substrate Ag atom^35^). The K/Ag(111) sample is immediately transferred after K deposition under UHV to the STM chamber and cooled for imaging. O_2_ vapor is dosed by admission through a variable leak valve onto a K/Ag(111) sample at ∼4.6 K temperature. In the absence of O_2_ dosing, adsorption of typical background gases under UHV (e.g., H_2_ or CO) is not observed.

### 2.2 Theory

To understand the interaction between the K atom and the O_2_ molecule on the Ag(111) surface, we perform DFT calculations by the Vienna ab initio simulation package (VASP) code.^36,37^ The core electrons are described by the projector augmented wave method.^38^ The exchange correlation potential is defined by the Perdew–Burke–Ernzerhof functional with the generalized gradient approximation.^39^ The geometry is optimized using a plane-wave cutoff energy of 500 eV and van der Waals correction of DFT-D3 with the Becke–Johnson damping method.^40,41^ The Brillouin zone is sampled in 3 × 3 grids with the Γ-point at the center. The energy and force values are converged with thresholds of 10^–6^ eV and 0.01 eV/Å, respectively. The Ag(111) surface is modeled by a $2\sqrt{3}\times 2\sqrt{3}$ supercell with a four-atomic-layer slab. For optimizing the K and O_2_ adsorption structure, the bottom two layers of the substrate are fixed at clean surface values. To avoid spurious interactions, the periodically repeated slabs are separated by 25 Å of vacuum. We use Bader charge analysis to evaluate the charge distribution of atoms in the system.^42–44^ This method determines the corresponding charge by calculating the Bader volume around each atom, effectively capturing the electronic transfer between different atoms. The energy profile along the reaction path is calculated by the nudged elastic band method, as implemented in the VASP transition state theory tools.^45^

## 3 Results and Discussion

### 3.1 O_2_ Adsorption on the K/Ag(111) Surface

Figure 1a presents the pristine Ag(111) surface, displaying two terraces separated by a monatomic step. The inset shows an atomically resolved STM topographic image of the same surface, revealing the characteristic hexagonal lattice of the Ag(111) substrate. A representative STM topographic image in Figure 1b shows ∼0.08 ML of K atoms deposited on the Ag(111) surface. Following K adsorption, hexagonally arranged bright protrusions cover the Ag(111) terraces, where each is identified as a single K adatom. K atoms assemble in a hexatic liquid structure on account of mutual dipole–dipole repulsion.^46,47^ The same superlattice is observed in different areas of the sample. Similar alkali adsorption characteristics have been reported for K on Au(111)^32^ and Cs on Ag(111).^47^

**Figure 1:**
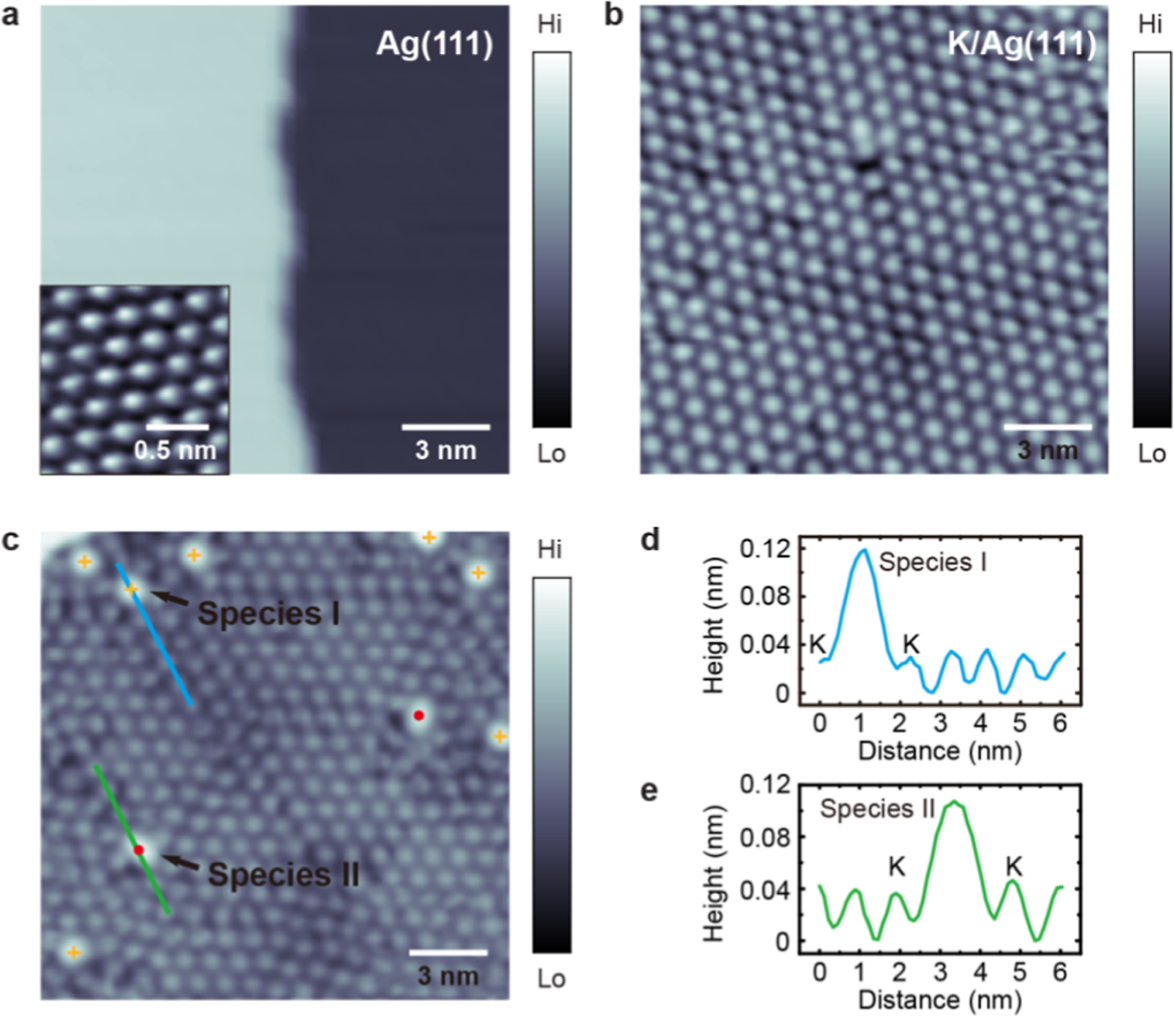
(a) STM topographic image of the Ag(111) surface at 4.6 K (+1.0 V, 100 pA, 16 × 16 nm^2^). The inset shows an atomically resolved STM topographic image of the pristine Ag(111) surface (+2.0 V, 100 pA, 1.5 × 1.5 nm^2^). (b) STM topographic image of K atoms on the Ag(111) surface at ∼0.08 ML coverage at 4.6 K (+1.0 V, 200 pA, 16 × 16 nm^2^). (c) STM topographic image after exposure of the K/Ag(111) surface to 0.36 L O_2_ at ∼4.6 K (+0.5 V, 100 pA, 18 × 18 nm^2^). Orange crosses and red dots mark species I and II, respectively. (d,e) Line profiles along the blue and green lines indicated in (c).

The STM image shown in Figure 1c is taken after exposure of the same K/Ag(111) surface to 0.36 Langmuir (L) of O_2_, but the scanning surface area is different from that in Figure 1b. O_2_ appears as occasional bright contrast involving interaction with the preadsorbed K atoms. Two structurally distinct bright adsorbate-induced images can be distinguished when the coverage is sufficiently low, so that most of the preadsorbed K atoms remain unaffected. Species I appear as bright round protrusions with six nearest neighbor K adatoms; seven such complexes are visible [orange crosses in Figure 1c]. A characteristic line profile [Figure 1d] of species I is taken along the blue line in Figure 1c. The measured separation from the bright protrusion to the two neighboring K adatoms is ∼1.13 nm, and their apparent height is ∼0.12 nm, as shown by the line profile [Figure 1d]. Considering that the measured average distance between the unreacted K adatoms for the present coverage is ∼1 nm, the stoichiometry within species I is likely to involve one oxygen molecule and one potassium atom forming a potassium superoxide (KO_2_) complex.

The O_2_-related feature species II in Figure 1c [red dots in Figure 1c] has a measured distance between the protrusion and two neighboring K adatoms of ∼1.45 nm [Figure 1e]. The larger distance compared to species I suggests that species II is a complex involving two potassium atoms, forming potassium peroxide (K_2_O_2_), thereby reducing the local density of unreacted K atoms. Given that our calculated oxygen dissociation barrier near a K atom on Ag(111) is 1.47 eV, with the O_2_ molecule positioned approximately 0.5 nm from the K adatom, and considering that our calculated O_2_ dissociation barrier on Ag(111) is 1.70 eV, it is unlikely that oxygen molecules dissociate into two oxygen atoms on the K/Ag(111) surface at 4.6 K, making the formation of the potassium oxide K_2_O complex highly improbable.^48^

The interaction of K with O_2_ on the Ag(111) surface can follow two distinct complexation processes leading to the potassium superoxide and peroxide species, as shown in Figure 2. The structural optimization presented in Figure 2 was performed using ionic relaxation techniques within the framework of density functional theory calculations. This allowed for an accurate determination of the system’s equilibrium geometry. The process involved adjusting the positions of ions to minimize total energy, with a convergence criterion ensuring that the forces on the ions were reduced to less than approximately 0.01 eV/Å. To model ∼0.08 ML atom coverage, the distance between K atoms occupying hollow sites of the Ag(111) surface is set to 0.99 nm [Figure S1]. Placing an O_2_ molecule at ∼0.54 nm from a K adatom leads to their attractive interaction, as shown in Figure 2a. The heights of adsorbates above the plane of the topmost layer of Ag atoms are 0.28 nm for K and 0.17 nm for the O_2_ molecules. Upon O_2_ adsorption, the preexisting reactant structures are optimized by minimizing the energy of the system, which causes the reaction complex to form without traversing an energy barrier, as depicted in Figure 2b. Prior to reaction, K atoms on the Ag(111) surface transfer 0.80 e^–^ of the valence 4s electron charge to the Ag(111) substrate [Figure S1c]. Upon complexation with O_2_, the K atom charge decreases from 0.20 e^–^ to 0.16 e^–^, while the O_2_ molecule charge increases from 0.39 e^–^ per atom to ∼0.54 e^–^ per atom in the complex [Figure S2a]. Upon forming the complex, the K atom is lifted up by 0.019 nm from 0.28 nm above the topmost Ag plane, while the O_2_ molecule is similarly lifted by 0.004 nm from 0.17 nm. The lifting of the complex with respect to the unreacted component structures is likely due to a reduction in their individual attractive image charges when the positive and negative reactants are brought together. Furthermore, charge transfer to the O_2_ molecule’s antibonding π* orbital weakens the O–O bond in KO_2_ compared to that in O_2_, causing its length to increase from 0.138 to 0.146 nm.

**Figure 2:**
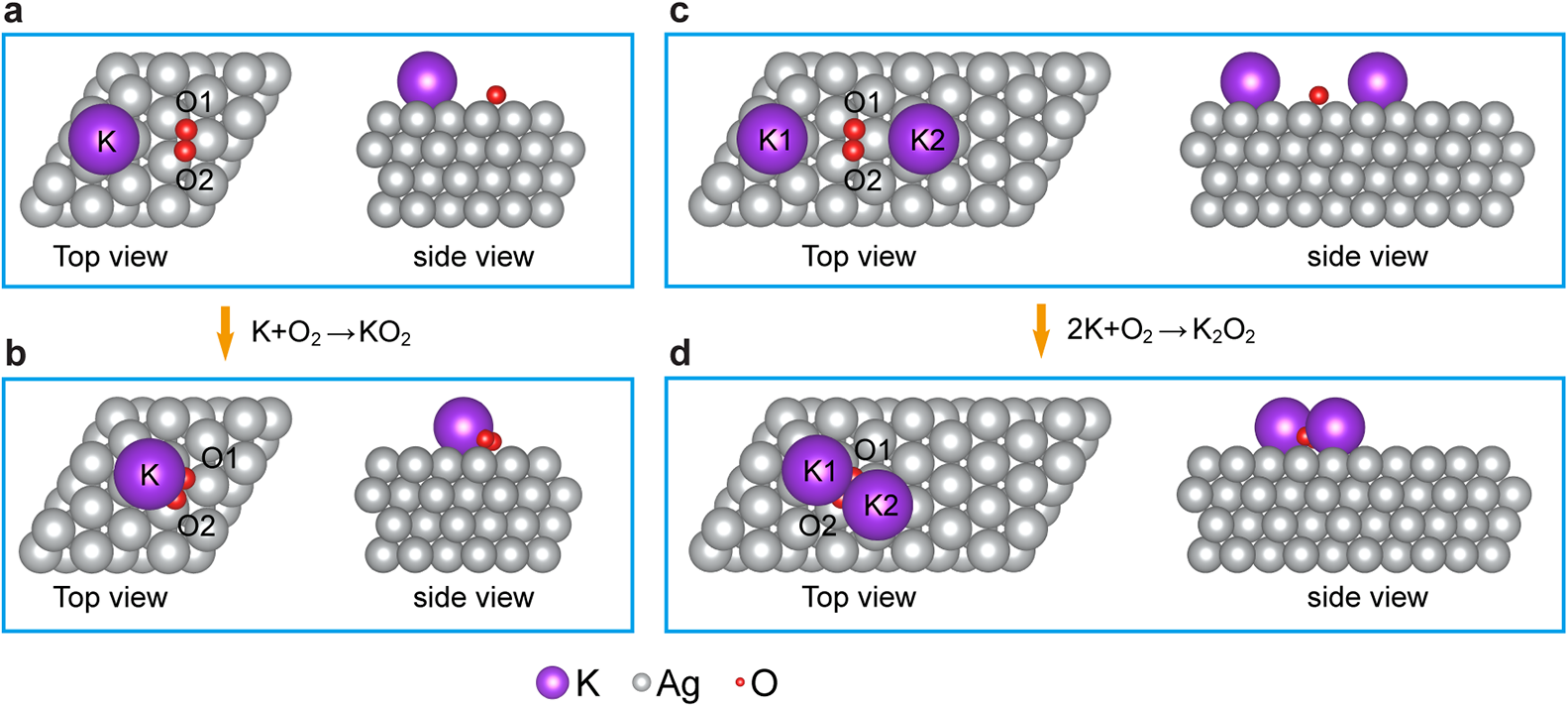
DFT calculation of the formation of KO_2_ (a,b) and K_2_O_2_ (c,d) complexes by the interaction of K adatoms and an O_2_ molecule on the Ag(111) surface. (a) Top and side views of the initial configuration of a K atom on the Ag(111) surface and an O_2_ molecule. (b) Top and side views of the optimized final configuration of the KO_2_ complex on the Ag(111) surface. (c) Top and side views of the initial configuration of two K atoms and an O_2_ molecule on the Ag(111) surface. (d) Top and side views of the optimized final configuration of the K_2_O_2_ complex on the Ag(111) surface.

Following the same procedure, we simulate the formation of a K_2_O_2_ complex. When an O_2_ molecule is placed between two K atoms [top and side views in Figure 2c], the optimized structure of the K–O_2_–K configuration is presented in Figure 2d. The charge transfer upon forming the K_2_O_2_ complex leaves 0.17 e^–^ on the K atom and ∼0.64 e^–^ per O atom on the O_2_ molecule [Figure S2b]. For K_2_O_2_, the charges gained by O atoms are calculated to be larger than that in KO_2_, because each K atom partially donates its electron to the two semifilled antibonding π* O_2_ orbitals. The reduced image–charge interaction between the K_2_O_2_ complex and the Ag(111) surface lifts K atoms by 0.012 nm and O atoms by 0.016 nm. Furthermore, both the K–O and O–O bond lengths in the K_2_O_2_ are larger than those in the KO_2_. The pertinent calculated parameters characterizing the KO_2_ and K_2_O_2_ complexes on the Ag(111) surface are summarized in Tables S1 and S2. It should be noted that exposure of the K/Ag(111) sample to O_2_ leads to a redistribution of charge, which modifies the interactions between neighboring K atoms. This modification may cause the local removal of the K atom-induced hexatic structure.

To better understand the interaction between different adsorption sites of K atoms and O_2_ molecules, we calculated the optimized structure of KO_2_ and K_2_O_2_ with the K atoms located at the other two distinct adsorption sites (top and bridge sites) on the Ag(111) surface. Our results indicate that the adsorption site of the K atoms does not influence the obtained stable structures of the KO_2_ [Figure S3] and K_2_O_2_ [Figure S4] on the Ag(111) substrate. We have performed additional DFT calculations to investigate the interaction of a single O_2_ molecule with an isolated K atom on the Ag(111) surface [Figure S5]. The optimized final configuration of the KO_2_ complex on the Ag(111) surface is consistent with that presented in Figure 2b, corresponding to a K atom coverage of approximately 0.08 ML.

From the calculated charges of the complex components, we conclude that the chemisorption-induced redox interaction transfers charge from K atoms to the O_2_ molecules, but the charge gained by the antibonding orbitals of the O_2_ molecule is larger than that lost by the K atoms, indicating that the Ag(111) substrate also has a role in the charge transfer process. This is reasonable because the chemisorption of O_2_ molecules on metals generally increases their work function, signifying metal-to-adsorbate charge transfer. Thus, the charge redistribution is defined by the Coulomb potentials of all three species, where the screening response of Ag(111) to the external charged species modulates both the ionic interaction and charge redistribution within the individual complexes.

To further investigate the structure of O_2_ interacting with K on the Ag(111) surface, we have measured the d*Z*/d*V* spectra and calculated the projected density of states (PDOS) for the K atom, KO_2_, and K_2_O_2_ on Ag(111), respectively. Figure 3a presents the d*Z*/d*V* spectra obtained for the K atom, KO_2_, and K_2_O_2_ on Ag(111). Notably, all three spectra exhibit a peak around +3.6 V, attributed to the image potential states.^49^ The peak at +2.1 V observed for the K/Ag(111) is in agreement with the K 4s state in the calculated PDOS and two-photon photoemission spectroscopy of the alkali atom-covered Ag(111) surface [Figure 3b].^50^ Significantly, this state is substantially perturbed by the O_2_ adsorption. The peak at +1.7 V is observed for KO_2_ on Ag(111), which is attributed to charge transfer from the K adatom to the antibonding π* orbital of the O_2_ molecule [Figure 3a]. By contrast, K_2_O_2_ exhibits an increased peak intensity and a shift in the peak position to +2.0 V compared with KO_2_, as shown in Figure 3a. This change is attributed to charge transfer from the two K adatoms to the O_2_ molecule. The calculation identifies a distinct feature in the PDOS for KO_2_ near +1.7 eV [Figure 3c] and for K_2_O_2_ near +2.0 eV [Figure 3d]. These calculated energy positions align well with the corresponding peaks observed experimentally at +1.7 V for KO_2_ and +2.0 V for K_2_O_2_ in the d*Z*/d*V* spectra [Figure 3a]. Meanwhile, the calculation reproduces the shift in the peak position between KO_2_ (+1.7 V) and K_2_O_2_ (+2.0 V). Figure 3e–h shows the structures of KO_2_ [Figure 3e] and K_2_O_2_ [Figure 3g] on the Ag(111) surface and their simulated STM images [Figure 3f and Figure 3h], which reproduce the experimental STM images of KO_2_ (bright round protrusion) and K_2_O_2_ (bright oval protrusion) in Figure 1c.

**Figure 3:**
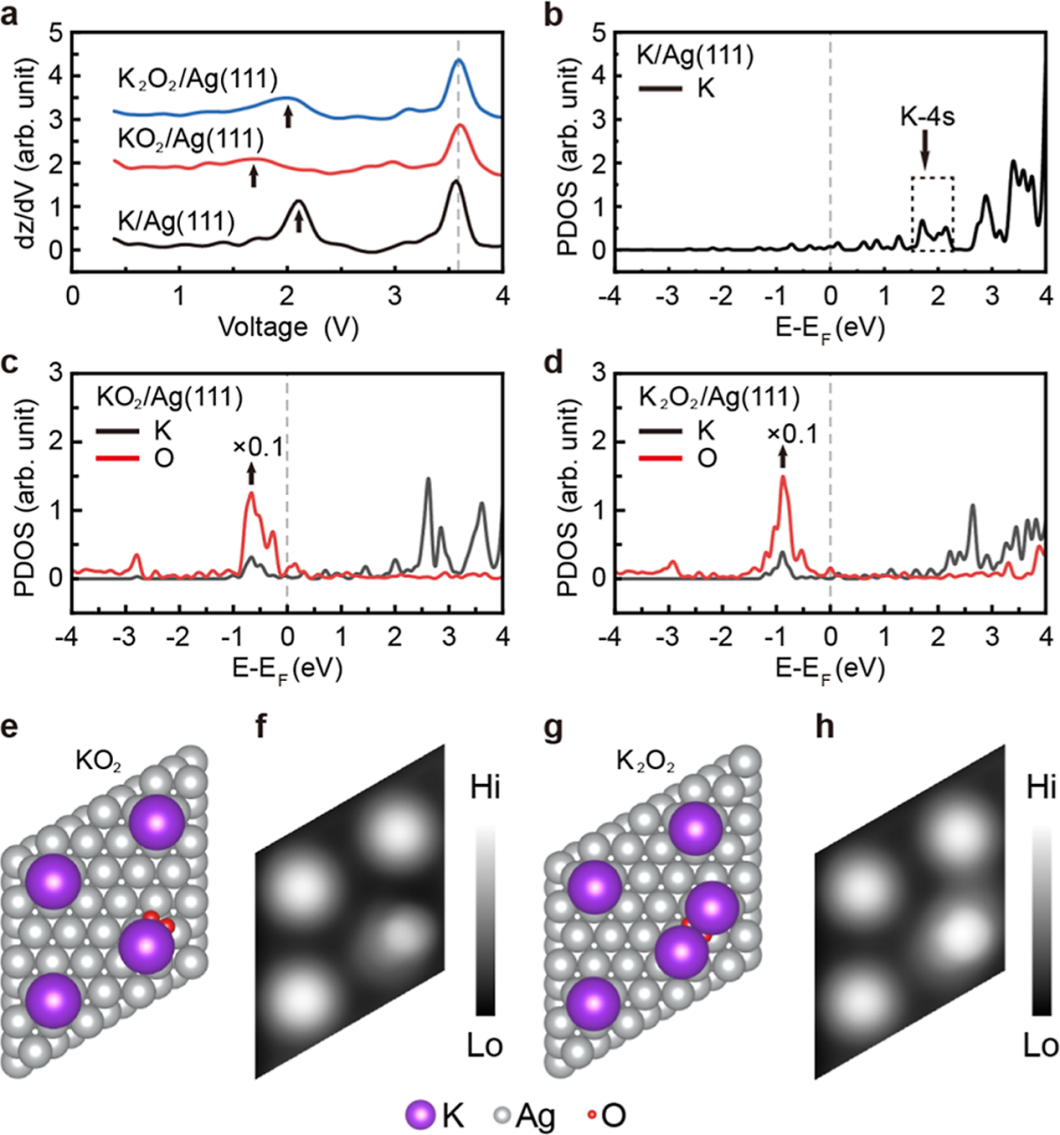
(a) d*Z*/d*V* spectra obtained for the K atom, KO_2_, and K_2_O_2_ on the Ag(111) surface. The arrows in (a) mark the experimental d*Z*/d*V* peaks observed at +2.1 V for the K atom, +1.7 V for KO_2_, and +2.0 V for K_2_O_2_. Calculated PDOS for the K atom (b), KO_2_ (c), and K_2_O_2_ (d) on the Ag(111) surface. The arrow in (b) points to a boxed region encompassing electronic states dominantly characterized by the K 4s orbital. The arrows in (c,d) indicate the calculated PDOS for oxygen in KO_2_ and K_2_O_2_, respectively. (e) Top view of the structure of the KO_2_ on the Ag(111) surface and (f) simulated STM topographic image of the KO_2_ on the Ag(111) surface. (g) Top view of the structure of the K_2_O_2_ on the Ag(111) surface and (h) simulated STM topographic image of the K_2_O_2_ on the Ag(111) surface.

To further understand the electronic interaction between O_2_ molecules and K atoms on the Ag(111) surface, we calculated the charge difference maps for the KO_2_ and K_2_O_2_ complexes on Ag(111) that are presented in Figure S6. For KO_2_ [Figure S6a,b], a depletion of charge density is observed around the potassium atom of the KO_2_ with charge accumulation observed around the O–O axis, which is related to the O_2_ molecule’s antibonding π* orbital. The induced charge density reveals that the potassium atom is cationic, and the oxygen atoms are anionic, as can be expected, leading to an attractive electrostatic interaction between them. Figure S6c,d shows the charge difference map for K_2_O_2_ on Ag(111), which similarly illustrates a depletion of charge density around two potassium atoms and an accumulation of charge around the O_2_ molecule. The accumulation of charge on the O_2_ molecule of K_2_O_2_, however, is greater than that for KO_2_ [Figure S2]. Hence, in both cases, the attraction between the K cations and the O_2_ anions in the KO_2_ and K_2_O_2_ complexes on Ag(111) is driven by the Coulomb forces.

### 3.2 Direct Observation of the Formation of KO_2_ and K_2_O_2_

The reduction of single O_2_ molecules by K atoms can be directly imaged by following the contrast of single K atoms changes upon exposure to O_2_ exposure. Figure 4a shows a typical STM topographic image with features attributed to three KO_2_ and nine K_2_O_2_ complexes on the Ag(111) surface, marked by dashed yellow circles and red dots, respectively. These reacted species are relatively stable, enabling the analysis of further interactions promoted by the adsorbed O_2_ molecules. The high mobility of K adatoms, arising solely from the weak corrugation of the Ag(111) surface that they experience, compromises atomic-scale resolution in STM imaging on some areas of the K/Ag(111) surface due to facile motion between nearly equivalent adsorption sites,^35^ as is evident in Figure 4. Figure 4b shows the same surface area as in Figure 4a following an additional exposure of the sample to 0.04 L of the O_2_ molecules. On comparison of the STM images before [Figure 4a] and after [Figure 4b] additional O_2_ exposure, we note that two new bright round-shaped protrusions (marked by dashed green circles) and one new bright oval-shaped protrusion (marked by a dashed blue circle) appear on the surface. Therefore, the sequential exposure to additional O_2_ molecules increases the number of both species. Based on our proposed reaction stoichiometries (K + O_2_ → KO_2_ and 2K + O_2_ → K_2_O_2_) and their shapes, these newly formed round and oval-shaped protrusions are recognized as KO_2_ and K_2_O_2_ complexes, respectively. These observations agree well with the STM measurements shown in Figure 1 and support the reaction mechanism where each new bright feature involves a single O_2_ molecule but does not perturb the existing KO_2_ and K_2_O_2_ complexes or introduce additional contrast from other unknown species [Figure 2].

**Figure 4:**
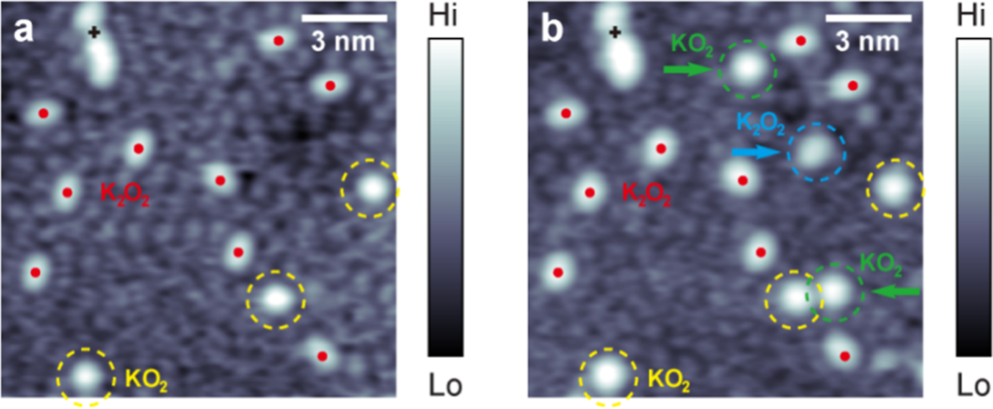
STM topographic images of the same area showing bright KO_2_ and K_2_O_2_ complexes on Ag(111) before (a) and after (b) dosing of additional 0.04 L O_2_ at ∼4.6 K. The preexisting KO_2_ and K_2_O_2_ complexes are marked by dashed yellow circles and red dots, respectively. The newly formed KO_2_ and K_2_O_2_ complexes are marked by dashed green and blue circles in (b), respectively. The new bright features show the same contrast as the preexisting complexes. An unidentified bright feature likely composed of several O_2_ molecules [marked by a black cross in (a) and (b)] serves as a stable position reference. Atomic-scale STM imaging of individual K adatoms in the K-covered regions is compromised by their high mobility on the Ag(111) surface, resulting in their inconsistent shapes. Scanning parameters: (a) +1.0 V, 100 pA, 14 × 14 nm^2^; (b) +1.6 V, 100 pA, 14 × 14 nm^2^.

To study further the interaction between the O_2_ molecules and K adatoms on the Ag(111) surface, we evaluate the statistical distribution of the bright features, individually assigned to the KO_2_ and K_2_O_2_ complexes. The statistical analysis is performed by counting the distinct contrast features in three large-scale STM images. Figure 5a presents an STM image of the K/Ag(111) surface after O_2_ exposure (0.36 L), where 8 KO_2_ complexes and 39 K_2_O_2_ complexes are visible. From such analysis of three different sampled parts of the surface, we extract the relative formation probability of the KO_2_ or K_2_O_2_ complexes from their population numbers. The results of this analysis are summarized in Figure 5b, with the yellow and red bars showing the formation counts of the KO_2_ and K_2_O_2_ complexes, respectively. Analysis of Figure 5a yields a composition of 17% KO_2_ and 83% K_2_O_2_, while the other two STM images [Figure S7] provide the additional statistics that are included in Figure 5b. Evidently, the formation of K_2_O_2_ complexes on the Ag(111) surface (∼78.1 ± 6.7%) is significantly more favored than that of KO_2_ complexes (∼21.9 ± 6.7%). Figure S8 presents an STM image of the K/Ag(111) surface after higher (1.8 L) exposure to O_2_. The subsequent STM image, however, displays aggregated cluster-like structures. Within these clusters, it becomes unreliable to identify KO_2_ and K_2_O_2_ or another distinctly different molecular species by STM.

**Figure 5:**
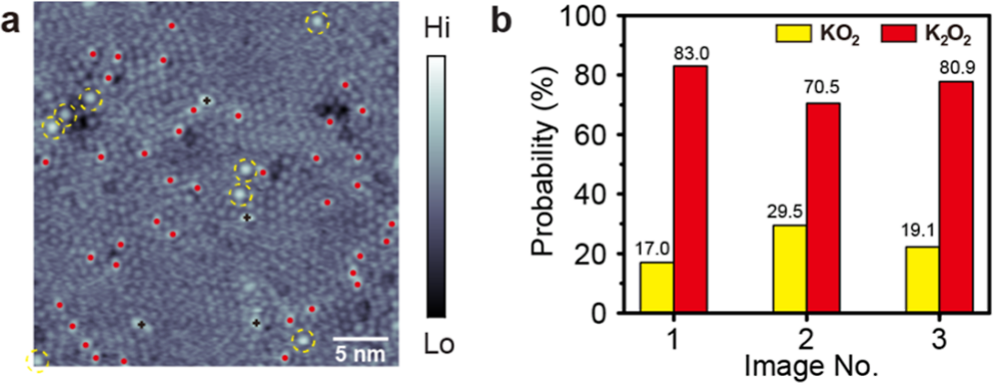
(a) A large-area STM topographic image after exposure of the K/Ag(111) surface to 0.36 L O_2_ at ∼4.6 K (+0.4 V, 100 pA, 33 × 33 nm^2^). The KO_2_ and K_2_O_2_ complexes are marked in (a) by dashed yellow circles and red dots, respectively. Four bright features of unknown origin are marked by black crosses in (a). (b) Formation probability of the KO_2_ and K_2_O_2_ complexes on the Ag(111) surface obtained from three large-area STM images. In the dominant (terminal) reaction, two K adatoms react with one O_2_ molecule, forming the K_2_O_2_ complex. The remaining bright spots are from the intermediate reaction where only one K atom has reacted, forming the KO_2_ complex.

In the context of Am–O_2_ batteries, it has been reported that the stability of the KO_2_ and K_2_O_2_ depends both on the temperature and O_2_ partial pressure.^15,51^ In the 0–500 °C temperature range, the K_2_O_2_ is the more thermodynamically stable phase at a lower O_2_ partial pressure, but the KO_2_ becomes more stable for increased O_2_ partial pressures.^52^ Recent scanning tunneling microscopy studies have demonstrated that the temperature plays a significant role in the formation of potassium oxides. After coadsorption of K and O_2_ on the Au(111) surface at 4.5 K, individual K_2_O_2_ complexes and K_2_O_2_ islands can form.^32^ Within the temperature range of 300–525 K, three different potassium oxides (K_2_O_2_, K_2_O, and KO_*y*_, where *y* < 0.5) were observed.^53^ In the present study, we only found KO_2_ and K_2_O_2_ complexes forming on Ag(111) at 4.6 K. The potentially informative effect of elevated temperature on coadsorption of O_2_ and K on the Ag(111) surface is a compelling topic for future research. In the potassium–oxygen batteries, the specific capacity of K_2_O_2_ is significantly higher than that of KO_2_.^54^ The formation of K_2_O_2_, however, has been shown to affect the fundamental metrices of energy storage, resulting in reduced Coulombic and lower round-trip efficiencies.^18^ Consequently, practical K–O_2_ cells are designed to prioritize the production of KO_2_ as the discharge product.^13^ Although the typical discharge conditions at low potentials do not favor the formation of K_2_O_2_, it can form through a chemical reaction on the carbon electrode surface, where KO_2_ is further reduced when the electrode is polarized at lower voltages.^55^ Additionally, it has been observed that the oxygen partial pressure influences the formation of discharge products in potassium–oxygen batteries, which usually operate at around 1 atm. Notably, the formation of K_2_O_2_ as a secondary reduction product of previously formed KO_2_ was observed when oxygen is completely removed from the K–O_2_ cell during discharge.^56^ In our experiments, the O_2_ vapor is dosed onto the sample surface at a low partial pressure, with the sample maintained at ∼4.6 K. We recognize at that temperature the surface is far from thermodynamic equilibrium. Therefore, the relative stability of K_2_O_2_ compared to KO_2_ is based primarily on the kinetic observations made at this low temperature rather than on the thermodynamic stability assessments.

### 3.3 Conversion from KO_2_ to K_2_O_2_ (KO_2_ + K → K_2_O_2_)

So far, we have described the stochastic formation of the KO_2_ and K_2_O_2_ complexes after the exposure of the K/Ag(111) surface to O_2_ at 4.6 K. The statistical analysis of the two products suggests that K_2_O_2_ complexes are more stable, as expected for a terminal reaction product. This observation is consistent with our previous STM study of coadsorption of O_2_ and K, but on the Au(111) surface, only the K_2_O_2_ complex was observed.^32^ Because the experiments are performed by the same procedures on the same instrument, we judge that the difference arises from different properties of the substrates. One can presume that the higher electron affinity of the Au substrate accepts more charge from K, making it more electropositive and therefore more reactive with the chemisorbed O_2_ molecules. Next, we experimentally investigated the relative stability of the KO_2_ and K_2_O_2_ complexes on the Ag(111) surface. Taking two consecutive STM images of the same area, we follow the persistence of the imaged KO_2_ and K_2_O_2_ complexes at 4.6 K. In the initial STM image [Figure 6a], we find five KO_2_ and eight K_2_O_2_ complexes marked as previously. Obvious changes in the bright structures are marked by dashed blue circles in the consecutive STM image of the same area in Figure 6b. From the comparison of the two STM images, we judge that three KO_2_ complexes have vanished, and instead, three additional K_2_O_2_ complexes have appeared over a 7 min period between the measurements. We interpret this observation by concluding that the KO_2_ complexes convert to K_2_O_2_ at ∼4.6 K under the STM tunneling conditions. A similar surface transformation is again observed in two consecutive STM images in Figure S9. The reverse transformation of K_2_O_2_ to KO_2_ has not been observed.

**Figure 6:**
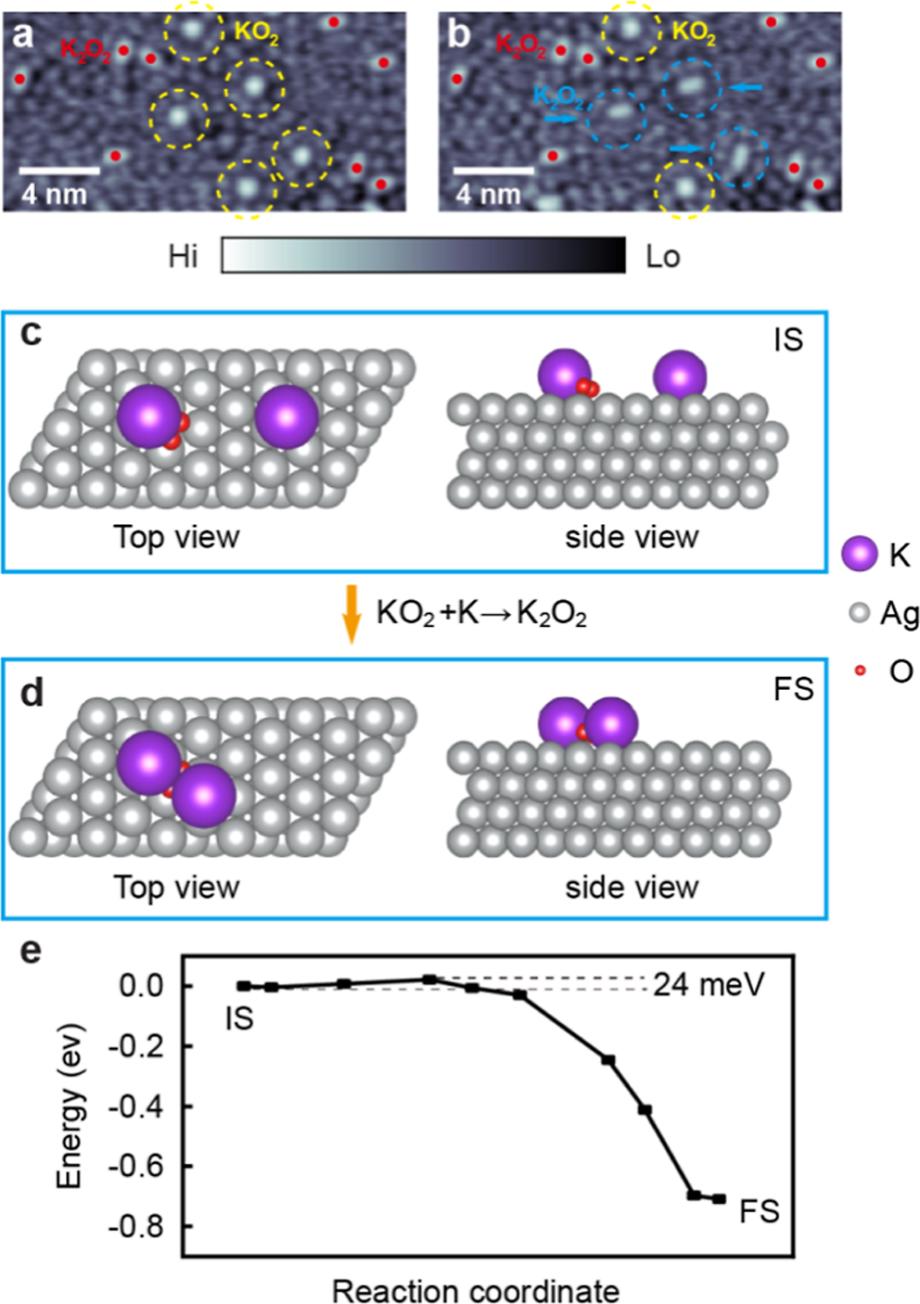
Formation of K_2_O_2_ via the KO_2_ + K → K_2_O_2_ reaction on Ag(111). (a,b) Two consecutive STM topographic images of the same area of the KO_2_ and K_2_O_2_ complexes on the Ag(111) surface. Scanning parameters: (a,b) +0.4 V, 100 pA, 20 × 10 nm^2^. The starting KO_2_ and K_2_O_2_ complexes are marked by dashed yellow circles and red dots in (a), respectively. The newly formed (after 7 min) K_2_O_2_ complexes are marked by dashed blue circles in (b). (c,d) DFT calculation of the formation of the K_2_O_2_ complex by addition of a second K atom to the KO_2_ complex on the Ag(111) surface. (c) Top and side views of the initial configuration of a KO_2_ complex and a proximate K atom on the Ag(111) surface. (d) Top and side views of the optimized final configuration of the K_2_O_2_ complex on the Ag(111) surface. (e) Nudged elastic band energy profile and barrier height for the formation of the K_2_O_2_ complex final state by addition of a second K atom to the KO_2_ complex.

To explain the experimental results presented above, we performed DFT calculations that addressed the formation of the K_2_O_2_ complex by the reaction of an additional K atom with a KO_2_ complex. The initial state (IS) and final state (FS) configurations are shown in Figures 6c and 6d, respectively. A K atom is placed near the optimized structure of the KO_2_ complex on the Ag(111) surface [Figure 6c], and the relaxed final structure is presented in Figure 6d. It is evident that the K atom moves toward the O_2_ molecule of the KO_2_ complex, forming the K_2_O_2_ product. Furthermore, the calculations show that the addition of a second K atom to the KO_2_ complex increases the O–O bond length in K_2_O_2_ by 0.04 to 1.501 Å, showing that the same structure is formed when two K atoms directly react with an O_2_ molecule on the Ag(111) surface [Figure 2c,d]. This is consistent with the experimental observations showing the same bright oval-shaped structure for the K_2_O_2_ complex, which is observed both promptly after O_2_ exposure and through conversion from the metastable KO_2_ complex in the proximity to the K atoms.

Figure 6e shows the energy profile calculated by using the nudged elastic band method for the formation of the K_2_O_2_ complex on Ag(111). The calculated barrier for the formation of the K_2_O_2_ complex by reaction with a second K atom to the KO_2_ complex is 24 meV. While the calculated activation energy suggests that the reaction could be facile, the reaction rate may be low under purely classical thermal activation at 4.6 K. It has been reported that the quantum tunneling effects could substantially enhance the reaction rates at very low temperatures, and prior studies have demonstrated that tunneling can enable reactions even when thermal activation may be negligible.^57,58^ Therefore, we tentatively interpret that the observed conversion from species I to II can be explained by a combination of thermal activation and quantum tunneling effects. Note that while the existence of this reaction has been postulated in a previous study of the KO_2_ growth on a glassy carbon electrode,^59^ here we confirm it by species-selective direct observations by atomic-scale STM imaging on Ag(111).

While our experiments are performed on isolated K–O_2_ complexes on an atomically flat Ag(111) surface at cryogenic temperature and thus differ from the bulk crystalline KO_2_ and K_2_O_2_ phases formed in practical K–O_2_ batteries, the insights gained are relevant to understanding the fundamental chemistry at electrode interfaces. In real battery cathodes, KO_2_ is generally the dominant discharge product, but it can undergo further chemical or electrochemical reduction to K_2_O_2_ under certain operating conditions such as low electrode potentials and reduced O_2_ partial pressure. The direct STM observation of KO_2_ → K_2_O_2_ conversion without an external O_2_ supply demonstrates that this transformation can occur via local surface reactions, consistent with mechanisms proposed in the battery studies. These atomic-scale observations provide mechanistic benchmarks for the nucleation and growth of alkali-oxygen phases at solid–electrolyte interfaces, where surface-mediated charge transfer, ion transport, and structural rearrangements play crucial roles. We emphasize that our findings do not capture electrolyte effects, morphological evolution, or the complexities of bulk-phase growth, which govern charging kinetics and cycle life in real devices. Nevertheless, isolating the elementary steps of the K–O_2_ interaction allows us to identify the intrinsic factors favoring superoxide versus peroxide formation, offering a framework for interpreting and potentially controlling discharge product distributions in working K–O_2_ electrodes.

Finally, it is important to note that we have not investigated the actual interaction behavior between potassium and oxygen molecules in practical potassium–oxygen batteries but rather focused on the prototypical K–O_2_ surface interactions and obtained some fundamental information regarding the coadsorption properties of O_2_ molecules and K atoms on metal surfaces. Further measurements are essential to evaluate the K–O_2_ surface chemistry under more realistic conditions for the battery to achieve a comprehensive understanding of how battery operation is affected by Am–O_2_ chemistry.

## 4 Conclusions

We have observed molecular O_2_-atomic potassium oxidation–reduction processes on the Ag(111) surface at the atomic level by low-temperature scanning tunneling microscopy. We show that the K and O_2_ interact on the Ag(111) surface to generate the distinct potassium superoxide, which irreversibly converts at 4.6 K on the Ag(111) surface to the more stable peroxide species. The attractive Coulomb interaction between the potassium cation and the O_2_ anion forms KO_2_ and K_2_O_2_ complexes on Ag(111). Our results reveal the chemical interactions of potassium atoms with molecular oxygen at the single-molecule level and provide fundamental information about the oxygen reduction chemistry, which is fundamentally important to understanding the mechanism of the O_2_ reaction in the K–O_2_ batteries. More broadly, we hope our study will support the development of the practical Am–O_2_ battery technology and facilitate a deeper understanding of the chemistry of single alkali metal cations, which play an important role in catalysis and surface chemistry.

## Supplementary Material



## Notes

Calculated structure model and charge difference map of K adsorption on the Ag(111) surface; calculated partial charges of K atoms and O_2_ molecules in KO_2_ and K_2_O_2_ complexes on the Ag(111) surface; DFT calculation of the formation of the KO_2_ complex by the interaction of a K adatom located at the bridge and top sites of the Ag(111) surface and an O_2_ molecule on the Ag(111) surface; DFT calculation of the formation of the K_2_O_2_ complex by the interaction of two K adatoms located at the bridge and top sites of the Ag(111) surface and an O_2_ molecule on the Ag(111) surface; DFT calculation of the formation of the KO_2_ complex by the interaction of an isolated K adatom with an O_2_ molecule on the Ag(111) surface; charge difference maps of the KO_2_ and K_2_O_2_ complexes on the Ag(111) surface; large-area STM topographic images after exposure of the K/Ag(111) surface to 0.36 L O_2_ at ∼4.6 K; STM topographic image after exposure of the K/Ag(111) surface (∼0.08 ML of K atoms) to 1.8 L O_2_ at ∼4.6 K; formation of K_2_O_2_ via the KO_2_ + K → K_2_O_2_ process on Ag(111); calculated structure parameters for the KO_2_ complex formed via the K + O_2_ → KO_2_ reaction on the Ag(111) surface; and calculated structure parameters for the K_2_O_2_ complex formed via the 2K + O_2_ → K_2_O_2_ reaction on the Ag(111) surface (PDF)

## References

[1] Li Y., Lu J. (2017). Metal–Air Batteries: Will They Be the Future Electrochemical
Energy Storage Device of Choice?. ACS Energy
Lett..

[2] Zubi G., Dufo-López R., Carvalho M., Pasaoglu G. (2018). The lithium-ion battery:
State of the art and future perspectives. Renew.
Sustain. Energy Rev..

[3] Thackeray M. M., Wolverton C., Isaacs E. D. (2012). Electrical energy storage for transportation—approaching
the limits of, and going beyond, lithium-ion batteries. Energy Environ. Sci..

[4] Iwaya K., Ogawa T., Minato T., Miyoshi K., Takeuchi J., Kuwabara A., Moriwake H., Kim Y., Hitosugi T. (2013). Impact of
Lithium-Ion Ordering on Surface Electronic States of Li_x_CoO_2_. Phys. Rev. Lett..

[5] Black R., Oh S. H., Lee J.-H., Yim T., Adams B., Nazar L. F. (2012). Screening for Superoxide Reactivity
in Li-O2 Batteries:
Effect on Li_2_O_2_/LiOH Crystallization. J. Am. Chem. Soc..

[6] Peng Z., Freunberger S. A., Hardwick L. J., Chen Y., Giordani V., Bardé F., Novák P., Graham D., Tarascon J.-M., Bruce P. G. (2011). Oxygen
Reactions in a Non-Aqueous Li^+^ Electrolyte. Angew. Chem., Int. Ed..

[7] Girishkumar G., McCloskey B., Luntz A. C., Swanson S., Wilcke W. (2010). Lithium–Air
Battery: Promise and Challenges. J. Phys. Chem.
Lett..

[8] Xiao N., Ren X., McCulloch W. D., Gourdin G., Wu Y. (2018). Potassium Superoxide:
A Unique Alternative for Metal–Air Batteries. Acc. Chem. Res..

[9] Choi J. W., Aurbach D. (2016). Promise and
reality of post-lithium-ion batteries with
high energy densities. Nat. Rev. Mater..

[10] Hartmann P., Bender C. L., Vračar M., Dürr A. K., Garsuch A., Janek J., Adelhelm P. (2013). A rechargeable
room-temperature
sodium superoxide (NaO_2_) battery. Nat. Mater..

[11] Qin L., Schkeryantz L., Zheng J., Xiao N., Wu Y. (2020). Superoxide-Based
K–O_2_ Batteries: Highly Reversible Oxygen Redox Solves
Challenges in Air Electrodes. J. Am. Chem. Soc..

[12] Aurbach D., McCloskey B. D., Nazar L. F., Bruce P. G. (2016). Advances
in understanding
mechanisms underpinning lithium–air batteries. Nat. Energy.

[13] Ren X., Wu Y. (2013). A Low-Overpotential
Potassium–Oxygen Battery Based on Potassium
Superoxide. J. Am. Chem. Soc..

[14] Park J., Hwang J.-Y., Kwak W.-J. (2020). Potassium–Oxygen Batteries:
Significance, Challenges, and Prospects. J.
Phys. Chem. Lett..

[15] Sangster J. (2013). K–O
(Potassium-Oxygen) System. J. Ph. Equilibria
Diffus..

[16] Wriedt H. A. (1987). The Na–O
(Sodium-Oxygen) System. Bull. Alloy Phase Diagr.

[17] Sangster J., Pelton A. D. (1992). The Li-O (lithium-oxygen) system. J. Ph. Equilibria.

[18] Wang W., Lu Y.-C. (2021). The Potassium–Air
Battery: Far from a Practical Reality?. Acc.
Mater. Res..

[19] Lu Y.-C., Gallant B. M., Kwabi D. G., Harding J. R., Mitchell R. R., Whittingham M. S., Shao-Horn Y. (2013). Lithium–oxygen batteries:
bridging mechanistic understanding and battery performance. Energy Environ. Sci..

[20] Lu Y. C., Xu Z., Gasteiger H. A., Chen S., Hamad-Schifferli K., Shao-Horn Y. (2010). Platinum-gold
nanoparticles: a highly active bifunctional
electrocatalyst for rechargeable lithium-air batteries. J. Am. Chem. Soc..

[21] Ren X. M., Zhang S. S., Tran D. T., Read J. (2011). Oxygen reduction reaction
catalyst on lithium/air battery discharge performance. J. Mater. Chem..

[22] Lu Y. C., Gasteiger H. A., Shao-Horn Y. (2011). Catalytic activity trends of oxygen
reduction reaction for nonaqueous Li-air batteries. J. Am. Chem. Soc..

[23] Dathar G.
K., Shelton W. A., Xu Y. (2012). Trends in the Catalytic Activity
of Transition Metals for the Oxygen Reduction Reaction by Lithium. J. Phys. Chem. Lett..

[24] Lamoreaux R. H., Hildenbrand D. L. (1984). High Temperature
Vaporization Behavior of Oxides. I.
Alkali Metal Binary Oxides. J. Phys. Chem. Ref.
Data.

[25] de
Paola R. A., Hoffmann F. M., Heskett D., Plummer E. W. (1987). The coadsorption
of oxygen and potassium on Ru(001): Evidence for the formation of
K–O compounds. J. Chem. Phys..

[26] Qiu S. L., Lin C. L., Chen J., Strongin M. (1990). Photoemission studies
of the low-temperature reaction of metals and oxygen. Phys. Rev. B.

[27] Baddorf A. P., Itchkawitz B. S. (1992). Identification
of oxygen species on single crystal
K(110). Surf. Sci..

[28] Garfunkel E. L., Somorjai G. A. (1982). Potassium and potassium oxide monolayers
on the platinum
(111) and stepped (755) crystal surfaces: A LEED, AES, and TDS study. Surf. Sci..

[29] Rocker G. H., Huang C., Cobb C. L., Redding J. D., Metiu H., Martin R. M. (1991). The interaction
of oxygen and potassium on the Ru(001)
surface. Surf. Sci..

[30] Hock K. M., Barnard J. C., Palmer R. E., Ishida H. (1993). Competing
routes for
charge transfer in co-adsorption of K and O_2_ on graphite. Phys. Rev. Lett..

[31] Bowker M., Grillo F., Archard D. (2019). CO and O_2_ Adsorption on
K/Pt(111). J. Phys. Chem. C.

[32] Ren J., Wang Y., Zhao J., Tan S., Petek H. (2019). K Atom Promotion
of O_2_ Chemisorption on Au(111) Surface. J. Am. Chem. Soc..

[33] Xu Y., Marbach H., Imbihl R., Kevrekidis I. G., Mavrikakis M. (2007). The Effect
of Coadsorbed Oxygen on the Adsorption and
Diffusion of Potassium on Rh(110): A First-Principles Study. J. Phys. Chem. C.

[34] Qiao Y., Deng H., Chang Z., Cao X., Yang H., Zhou H. (2021). A high-capacity cathode for rechargeable
K-metal battery based on
reversible superoxide-peroxide conversion. Natl.
Sci. Rev..

[35] Zhang C., Chen L., Zhao J., Petek H. (2022). Imaging a
Haber-Bosch
catalysis precursor at the atomic scale. Cell
Rep. Phys. Sci..

[36] Kresse G., Hafner J. (1993). Ab initio molecular
dynamics for open-shell transition
metals. Phys. Rev. B.

[37] Kresse G., Furthmuller J. (1996). Efficiency of *Ab-Initio* Total Energy
Calculations for Metals and Semiconductors Using a Plane-Wave Basis
Set. Comput. Mater. Sci..

[38] Blöchl P. E. (1994). Projector
augmented-wave method. Phys. Rev. B.

[39] Perdew J. P., Burke K., Ernzerhof M. (1996). Generalized
Gradient Approximation
Made Simple. Phys. Rev. Lett..

[40] Grimme S., Antony J., Ehrlich S., Krieg H. (2010). A consistent and accurate
ab initio parametrization of density functional dispersion correction
(DFT-D) for the 94 elements H-Pu. J. Chem. Phys..

[41] Grimme S., Ehrlich S., Goerigk L. (2011). Effect of the damping function in
dispersion corrected density functional theory. J. Comput. Chem..

[42] Henkelman G., Arnaldsson A., Jónsson H. (2006). A fast and robust algorithm for Bader
decomposition of charge density. Comput. Mater.
Sci..

[43] Sanville E., Kenny S. D., Smith R., Henkelman G. (2007). Improved grid-based
algorithm for Bader charge allocation. J. Comput.
Chem..

[44] Tang W., Sanville E., Henkelman G. (2009). A grid-based
Bader analysis algorithm
without lattice bias. J. Phys.: Condens. Matter.

[45] Henkelman G., Uberuaga B. P., Jónsson H. (2000). A climbing
image nudged elastic band
method for finding saddle points and minimum energy paths. J. Chem. Phys..

[46] Diehl R. D., McGrath R. (1996). Structural studies
of alkali metal adsorption and coadsorption
on metal surfaces. Surf. Sci. Rep..

[47] Ziegler M., Kröger J., Berndt R., Filinov A., Bonitz M. (2008). Scanning tunneling
microscopy and kinetic Monte Carlo investigation of cesium superlattices
on Ag(111). Phys. Rev. B.

[48] Halverson F. (1962). Comments on
potassium superoxide structure. J. Phys. Chem.
Solids.

[49] Dougherty D. B., Maksymovych P., Lee J., Feng M., Petek H., Yates J. T. (2007). Tunneling spectroscopy
of Stark-shifted image potential
states on Cu and Au surfaces. Phys. Rev. B.

[50] Wang L.-M., Sametoglu V., Winkelmann A., Zhao J., Petek H. (2011). Two-Photon
Photoemission Study of the Coverage-Dependent Electronic Structure
of Chemisorbed Alkali Atoms on a Ag(111) Surface. J. Phys. Chem. A.

[51] Qin L., Ao H., Wu Y. (2024). Feasibility
of achieving two-electron K–O_2_ batteries. Faraday Discuss..

[52] Oliver, G. Defect Chemistry in Alkali Peroxides and Superoxides. Ph.D. Thesis, Universität Stuttgart, **2014**.

[53] Shi R., Liao W., Ramirez P. J., Orozco I., Mahapatra M., Kang J., Hunt A., Waluyo I., Senanayake S. D., Liu P. (2022). The Interaction of K and O_2_ on Au(111):
Multiple Growth Modes of Potassium Oxide and Their Catalytic Activity
for CO Oxidation. Angew. Chem., Int. Ed..

[54] Küpper J., Simon U. (2022). The effects of oxygen pressure on
the discharge performance of potassium–oxygen
batteries. Sustainable Energy & Fuels.

[55] Sankarasubramanian S., Ramani V. (2018). Dimethyl Sulfoxide-Based Electrolytes for High-Current
Potassium–Oxygen Batteries. J. Phys.
Chem. C.

[56] Wang W., Wang Y., Wang C.-H., Yang Y.-W., Lu Y.-C. (2021). In Situ
probing of solid/liquid interfaces of potassium–oxygen batteries
via ambient pressure X-ray photoelectron spectroscopy: New reaction
pathways and root cause of battery degradation. Energy Storage Mater..

[57] Hazra J., Balakrishnan N. (2015). Quantum dynamics
of tunneling dominated reactions at
low temperatures. New J. Phys..

[58] Meisner J., Kästner J. (2016). Atom Tunneling in Chemistry. Angew. Chem., Int. Ed..

[59] Wang W., Lai N.-C., Liang Z., Wang Y., Lu Y.-C. (2018). Superoxide
Stabilization and a Universal KO_2_ Growth Mechanism in Potassium–Oxygen
Batteries. Angew. Chem., Int. Ed..

