# Supplementary material for: An Atomic-Scale Mechanism of Potassium–Oxygen Redox Chemistry
Source: JACS Au. 2025 Sep 5;5(9):4530–8. doi: 10.1021/jacsau.5c00855 (PMC12458050; doi:10.1021/jacsau.5c00855)
Supplement: Supplementary file 1 [file au5c00855_si_001.pdf]

Supporting Information

Atomic-scale Mechanism of Potassium-oxygen Redox Chemistry

Chao Zhang,[§,||] Linjie Chen,[‡,||] Jin Zhao,[§] and Hrvoje Petek*,[†]

[§]Hefei National Research Center for Physical Sciences at the Microscale, University of Science and Technology of China, Hefei, Anhui 230026, China

[‡]Department of Chemical Physics, School of Chemistry, University of Science and Technology of China, Hefei, Anhui 230026, China

[†]Department of Physics and Astronomy and the IQ Initiative, University of Pittsburgh, Pittsburgh, Pennsylvania 15260, USA

Email: petek@pitt.edu

Contents

1. Supporting Figures...2

2. Supporting Tables...11

1. Supporting Figures

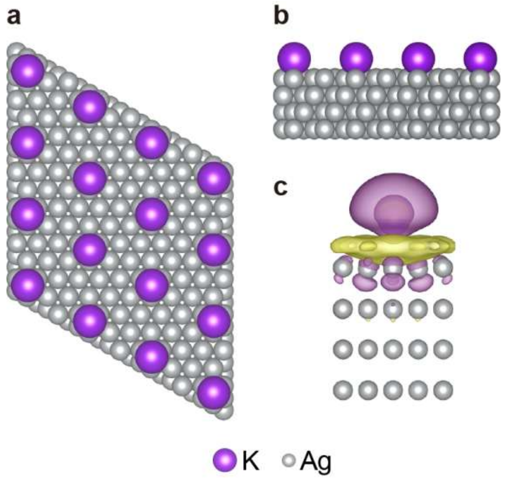

Figure S1. Top (a) and side views (b) of the calculated structure model of K adatoms on Ag(111) surface. For the calculated coverage of ～0.08 ML, the distance between the K adatoms is 0.99 nm, and they occupy hollow sites of the Ag(111) surface. This coverage approximately corresponds to that of the STM measurements. (c) Charge difference map for K adatom on Ag(111) surface. The yellow and purple isosurfaces represent the accumulation and depletion of electron density, respectively.

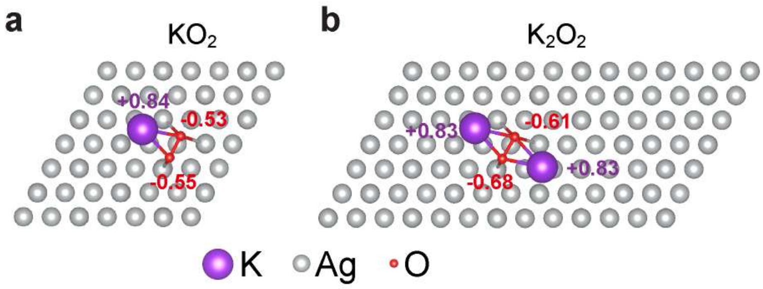

Figure S2. Calculated partial charges of K atoms and O_2 molecules in KO_2 (a) and K_2O_2 (b) complexes on Ag(111) surface calculated by a Bader charge analysis.

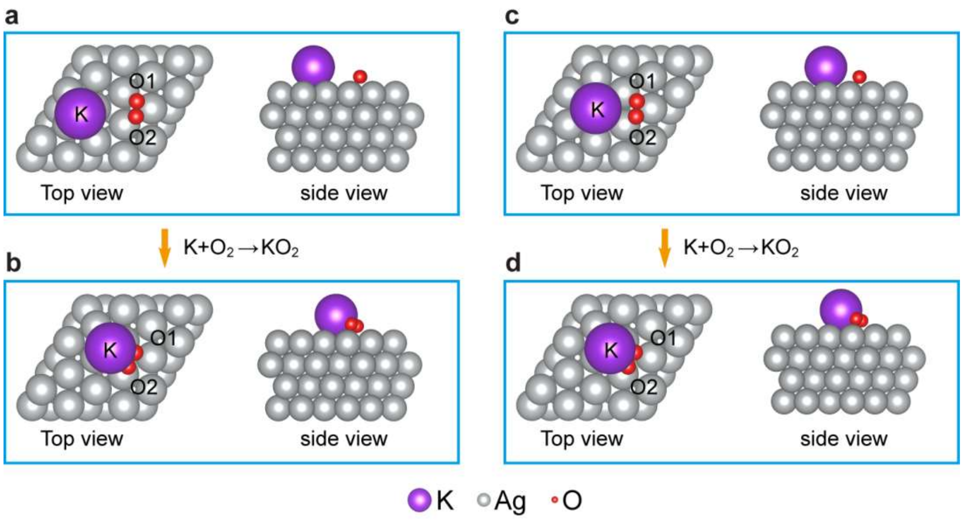

Figure S3. DFT calculation of the formation of KO_2 complex by the interaction of a K adatom and an O_2 molecule on Ag(111) surface. (a) Top and side views of the initial configuration of a K atom located at the bridge site of the Ag(111) surface and an O_2 molecule. (b) Top and side views of the optimized final configuration of the KO_2 complex on Ag(111) surface. (c) Top and side views of the initial configuration of a K atom located at the top site of the Ag(111) surface and an O_2 molecule. (d) Top and side views of the optimized final configuration of the KO_2 complex on Ag(111) surface.

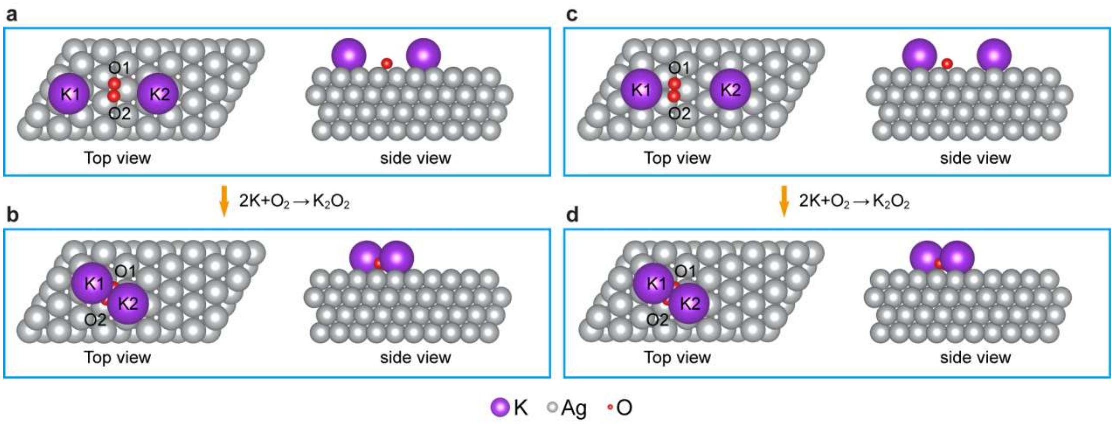

Figure S4. DFT calculation of the formation of K_2O_2 complex by the interaction of two K adatoms and an O_2 molecule on Ag(111) surface. (a) Top and side views of the initial configuration of two K atoms located at bridge sites of the Ag(111) surface and an O_2 molecule. (b) Top and side views of the optimized final configuration of the K_2O_2 complex on Ag(111) surface. (c) Top and side views of the initial configuration of two K atoms located at top sites of the Ag(111) surface and an O_2 molecule. (d) Top and side views of the optimized final configuration of the K_2O_2 complex on Ag(111) surface.

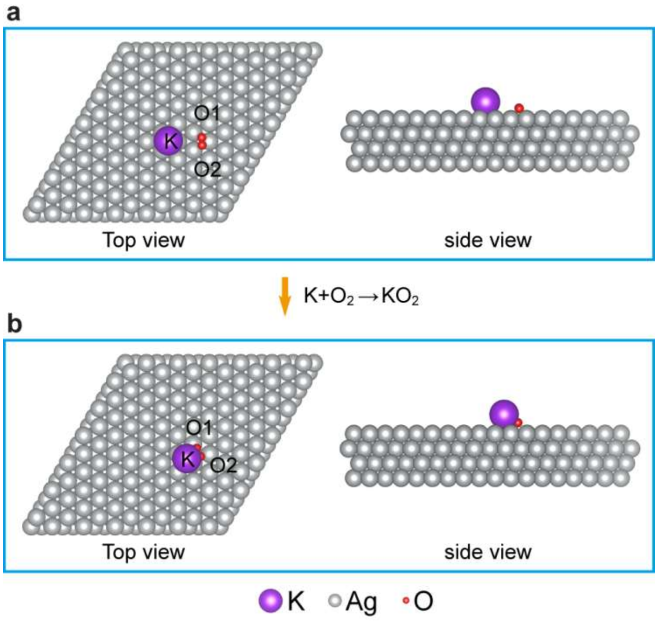

Figure S5. DFT calculation of the formation of KO_2 complex by the interaction of an isolated K adatom with an O_2 molecule on Ag(111) surface. (a) Top and side views of the initial configuration of an isolated K atom on Ag(111) surface and an O_2 molecule. (b) Top and side views of the optimized final configuration of the KO_2 complex on Ag(111) surface.

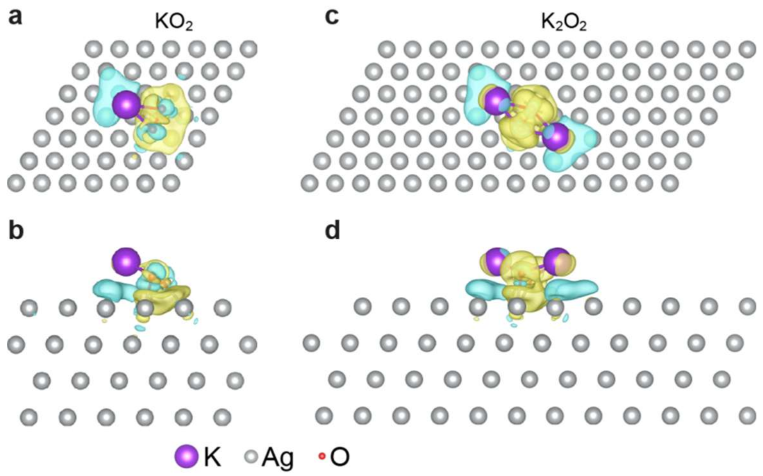

Figure S6. Charge difference maps of KO_2 and K_2O_2 complexes on the Ag(111) surface. Upper (a, c) and lower (b, d) panels show top and side views of each configuration. The yellow and cyan isosurfaces represent the accumulation and depletion of electron density, respectively. The O_2 charge accumulation clearly reflects the occupation of the π^* antibonding state. The charge density difference is calculated from $\Delta\rho = \rho_{K+O_2/Ag(111)} - (\rho_{K/Ag(111)} + \rho_{O_2})$, where $\rho_{K+O_2/Ag(111)}$ is the total charge density when O_2 and K are coadsorbed on Ag(111), while $\rho_{K/Ag(111)}$ and ρ_{O_2} represent the charge densities of K adsorbed on the Ag(111) and the O_2 molecule, respectively.

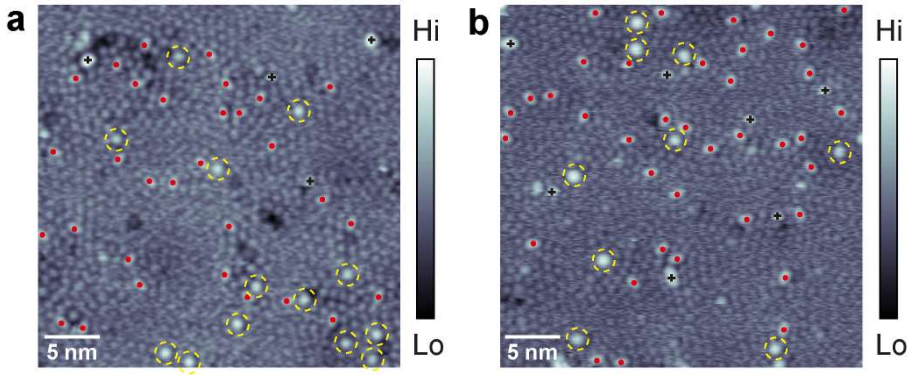

Figure S7. Large area STM topographic images after exposure of the K/Ag(111) surface to 0.36 L O_2 at ~4.6 K. The KO_2 and K_2O_2 complexes are marked by dashed yellow circles and red dots in (a) and (b), respectively. The unknown bright features are marked by black crosses in (a) and (b). Scanning parameters: (a) +0.4 V, 100 pA, 33×33 nm^2, (b) +1.0 V, 100 pA, 33×33 nm^2.

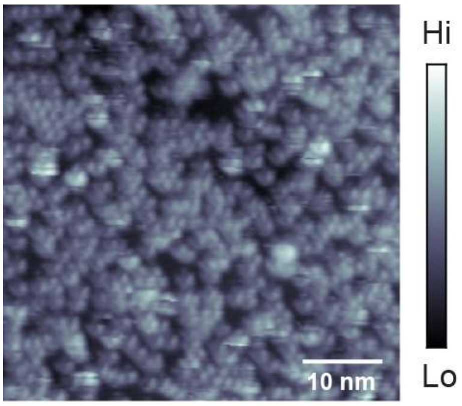

Figure S8. STM topographic image after exposure of the K/Ag(111) surface (~0.08 ML of K atoms) to 1.8 L O_2 at ~4.6 K (+1.0 V, 200 pA, 50×50 nm^2).

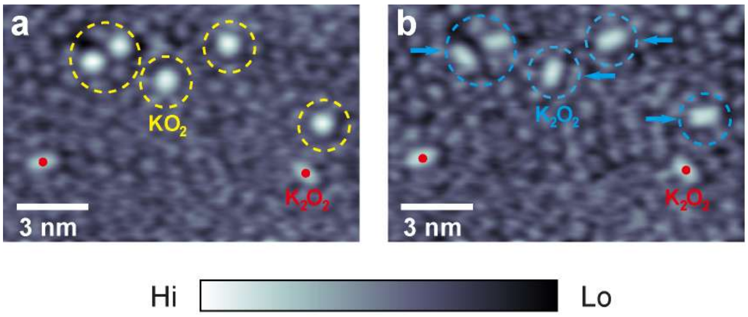

Figure S9. Formation of K_2O_2 via the KO_2+K→K_2O_2 process on Ag(111). (a) and (b) Two consecutive STM topographic images of the same area of the KO_2 and K_2O_2 complexes on Ag(111) surface. Scanning parameters: (a, b) +0.4 V, 100 pA, 15×10 nm^2. The KO_2 and K_2O_2 complexes are marked by dashed yellow circles and red dots in (a), respectively. The newly formed three K_2O_2 complexes are marked by dashed blue circles in (b).

2. Supporting Tables

$\mathbf{R}_{K-O1}$ (Å)	$\mathbf{R}_{K-O2}$ (Å)	$\mathbf{R}_{O1-O2}$ (Å)	∠O1KO2 (deg)
2.569	2.496	1.461	33.505

Table S1. Calculated structural parameters for KO_2 complex formed via $K+O_2 \rightarrow KO_2$ reaction on Ag(111) surface.

$\mathbf{R}_{K1-O1}$ (Å)	$\mathbf{R}_{K1-O2}$ (Å)	$\mathbf{R}_{K2-O1}$ (Å)	$\mathbf{R}_{K2-O2}$ (Å)	$\mathbf{R}_{O1-O2}$ (Å)	∠O1K1O2 (deg)	∠O1K2O2 (deg)
2.593	2.517	2.596	2.513	1.501	34.124	34.120

Table S2. Calculated structural parameters for K_2O_2 complex formed via $2K+O_2 \rightarrow K_2O_2$ reaction on Ag(111) surface.
